# COVID-19 in Fabry disease: a reference center prospective study

**DOI:** 10.1186/s13023-022-02386-7

**Published:** 2022-06-28

**Authors:** Christina Bothou, Lanja Saleh, Arnold von Eckardstein, Felix Beuschlein, Albina Nowak

**Affiliations:** 1 Department of Endocrinology, Diabetology and Clinical Nutrition, University Hospital Zurich (USZ) and University of Zurich (UZH), Rämistrasse 100, 8091 Zurich, Switzerland; 2 Department of Laboratory Medicine, University Hospital Zurich, Zurich, Switzerland; 3 Department of Internal Medicine, Psychiatry University Hospital Zurich, Zurich, Switzerland

**Keywords:** Fabry disease, Coronavirus disease 2019 (COVID-19), SARS-CoV-2 Antibody Titer

## Abstract

**Background:**

During the coronavirus disease-19 (COVID-19) pandemic, vulnerable populations must be identified to prevent increased mortality. Fabry disease (FD) is a rare X-linked lysosomal storage disorder leading to chronic kidney disease (CKD), cardiomyopathy, pneumonopathy and premature strokes. Little is known whether SARS-CoV-2 infection bears a particular risk for FD patients.

**Methods:**

During pandemic (02.2020–03.2021) we have regularly followed 104 unvaccinated FD patients. In 61/104, titre of serum antibodies against SARS-CoV-2 were measured and SARS-CoV-2 PCR test was performed in symptomatic patients or in case of positivity of other family members. The symptoms and duration of COVID-19 were reported by the patients or the treating physician.

**Results:**

No deaths or intensive care unit hospitalizations occurred. 13/104 (12.5%) were diagnosed with SARS-CoV-2 infection (16.7% (4/24) men 12.2% (6/49) women of classic phenotype, 25% (3/12) of the men and 0% (0/8) of the women of later- onset phenotype). Of those, 2/13 (15.4%) patients—both kidney transplant recipients—developed severe COVID-19, were hospitalized, and required a high-flow oxygen mask. The rest either developed mild COVID-19 manifestations (8/13, 61.5%) or were asymptomatic (3/13, 23.1%). 2/13 (15.4%) of the patients experienced Fabry pain crisis and 3/13 (23.1%) long COVID-19 like symptoms.

**Conclusions:**

Similar to the general population, in FD patients the risk for severe COVID-19 seems to be driven by the immune system rather than by FD itself. Immunosuppression in kidney transplant recipients represented the highest risk in this population.

**Supplementary Information:**

The online version contains supplementary material available at 10.1186/s13023-022-02386-7.

## Background

One of the lessons we have learned during the coronavirus disease- 19 (COVID-19) pandemic is that vulnerable populations must be identified and treated early, to reduce morbidity and mortality. Additionally, detailed assessment of the immunological status and response of certain patient populations could, on one hand, provide useful pathophysiological insights and shed light to unknown disease mechanisms and, on the other hand, indicate appropriate management and therapeutic strategies [1].

Fabry disease (FD) is a rare X-linked glycosphingolipid storage disease caused by mutations in the α- galactosidase A gene (*GLA*), which leads to reduced activity of the encoded lysosomal α-galactosidase A enzyme (α-Gal A). The α-Gal A defect causes the progressive accumulation of glycosphingolipid, and especially globotriaosylceramide (Gb3), in body liquids and tissue lysosomes [2]. There are two clinical phenotypes, classic and later-onset. Most of the patients with classic phenotype, especially males, suffer from the initial symptoms of the disease already in childhood due to little or no residual a-Gal A activity. More specifically, intracellular Gb3 accumulations lead to a variety of signs and symptoms such as acroparaesthesias, angiokeratoma, gastrointestinal cramping and diarrhoea, hypohidrosis and corneal dystrophy [3]. Inflammation and fibrosis, following Gb3 accumulations, lead to disease progression with development of kidney and heart failure, cerebrovascular disease, lung problems and, ultimately, early mortality [4, 5]. In contrast, patients with later-onset phenotype have residual α-Gal A activity, they lack the early manifestations seen in classic Fabry patients: males present in adulthood with cardiac or, less frequently, kidney disease, while females experience a milder disease course [6]. In general, FD females can have a variable phenotypic expression, from asymptomatic to severe, which is primarily associated with skewed X-chromosomal inactivation [7]. Over the last years, intravenously administered enzyme replacement therapy (ERT) with agalsidase alpha or beta or with oral chaperone therapy have been approved for the treatment of patients with FD and benefit the disease course [8]. Despite these therapies, FD patients still suffer from disease complications, partly because the effectiveness of the treatment varies among patients and partly due to irreversible organ damage already present at the time of diagnosis [9, 10].

COVID-19 primarily affects the respiratory system, but can involve all organs in the complicated form. Thus, as multisystemic disease, FD could be vulnerable population for severe COVID-19. Therefore, and assuming higher risk versus the general population, early in the pandemic, FD expert recommendations enforced special consideration for patients with FD [11]. However, at that time point, real-life FD population studies were missing to confirm the potential vulnerability and indicate prognostic factors for severe infection.

We aimed to investigate, whether FD patients are at higher risk of developing severe COVID-19 with unfavorable complications such as mortality and hospitalization, and to identify its risk factors. Of note, we conducted this observational study during the pre-vaccination time and assessed a complete cohort with no loss to follow-up. We have screened a large number of patients for SARS-CoV2 antibodies, and we have conducted polymerase chain reaction (PCR) diagnostics in symptomatic patients and for contact tracing in all reported cases.

## Methods

### Patients and methods

Overall, 104 FD patients (24 classic and 12 later- onset phenotype men and 49 classic and 19 later- onset phenotype women, mean ages 41.71 ± 13.51, 54.25 ± 15.04 and 42.53 ± 16.75 and 39.21 ± 16.80 respectively) were treated and followed at the specialized Fabry center between February 1 2020 and March 30 2021. Written informed consent was obtained from all patients, in accordance with the Declaration of Helsinki of 1975 as revised in 2000. The central ethical Committee of the University of Zurich approved this study and by the Zurich Ethics Committee (KEK-ZH-Nr. 2014-0534 and 2021-01958).

All patients had a pathogenic *GLA*-variant. The pathogenicity and phenotyping of the variants is shown in Additional file 1: Table S1 and is based on genotype and residual α‐Gal A activity in males as published in the International Fabry Disease Genotype/Phenotype Database (http://www.dbFGP.org) for all mutations of this population.

At the beginning of the pandemic, the treating physician provided instructions how to reduce risk of infection with SARS-CoV-2 by educational outreach calls or personal consultation of patients. All patients were counselled to adhere to the general guidelines and recommendations for avoiding an infection, including hand hygiene, social distancing, face mask use, restriction of the time that they stay outside the house and in public places, if possible, home-office, in case of symptoms, immediate testing and self-isolation, in case of disease of other family member, strictly follow the in-house isolation recommendations. ERT was administered via specialized home-care nursing staff in all patients, which has been a common practice for most patients already before pandemic. For the administrations and the nurse- patient contact, the standard hand-hygiene as well as the use of FFP2/N95 masks were strictly followed. Moreover, patients and the responsible nurses were instructed to contact the Fabry center in the case of positive SARS-CoV-2 testing of the patients, COVID-19- typical symptoms and for hospitalizations.

During the observational period, the Fabry center continued offering clinical patient visits that included COVID-19- history and blood sampling for measuring of SARS-CoV-2 IgG antibody levels. Serum samples were centrifuged and immediately frozen at − 80 °C, until later batch analysis. The SARS-CoV-2 IgG antibody levels were determined by applying the commercial Elecsys Anti-SARS-CoV-2 assay (reagent Lot number: 52691600, Roche Diagnostics, Mannheim, Germany) to a Cobas e802 immunoassay analyser. One sample of each patient was used. The determination method was qualitative. All measurements were carried out according to the manufacturer’s instructions at the Institute of Clinical Chemistry, University Hospital of Zurich. Clinical information on a possible SARS-CoV-2 infection regarding symptoms and their duration were acquired individually for each patient. Overall, 61 out of the 104 patients have visited our department during the observatory period and blood samples were drawn from them while the rest were followed up via telemedicine according to routine or more frequently if needed.

Patients who developed COVID-19-typical symptoms were advised to undergo naso-pharyngeal PCR testing. The results were reported to the Fabry center by the patients themselves, home care nurses or family doctors.

Patients were categorized into risk groups for severe COVID-19 according to the Fabry-related organ manifestations, based on a modified Fabry- specific risk stratification originally suggested by the international expert recommendations (Table 1) [11].

**Table 1:** Modified Fabry- specific risk stratification for severe COVID-19 originally suggested by the international expert recommendations [11]

Condition	Risk category
Patients with uncomplicated FD No existing severe organ damage at heart, lungs, kidney, and CNS	A
Patients with advanced FD Patients carrying at least one of the following: I. CKD (defined as pathological albuminuria > 30 mg/g urinary creatinine or eGFR < 60 mL/min/1.73m2) II. Heart involvement: arrhythmia or severe LVH or cardiomegaly III. CNS involvement: Stroke or TIA or WML IV. Pulmonary involvement: FEV1 decrease V. Age older than 40 years for classic males or older than 55 years for all other disease types	B
Patients with transplantation	C

FD: Fabry Disease; CNS: Central nervous system; CKD: chronic kidney disease; eGFR: estimated Glomerular Filtration Rate; LVH: Left Ventricular Hypertrophy; TIA: Transient Ischemic Attack; WHL: White Matter Lesion; FEV1: Forced Expiratory Pressure in 1 Second

All patients were regularly screened for high blood pressure and chronic kidney disease including the CKD stage during the annual examinations. In total, 3/102 active patients of our cohort received a kidney transplant and were under immunosuppression therapy. No other immunosuppressed patient including steroid treatment belongs to our cohort. The patients are in treatment (presented in median with interquartile range -IQR) with ERT for the following duration in years (men classic phenotype: 17.5 [9, 19], women classic phenotype: 10 [7.5,10.5], later- onset phenotype men 11 [7, 14], no later- onset phenotype woman treated with ERT) and with chaperone for the following duration in years (no men of classic phenotype treated with chaperone, women classic phenotype: 2.65 [1.3,4], later- onset phenotype men 3 [2.75,3.25] and later- onset phenotype woman 4). Overall, the patients of the whole cohort based on the complications and the age- criteria as presented in Table 1 were categorized in the following risk groups for severe COVID-19: men of classic phenotype 2A, 20B, 2C (n = 24), women classic phenotype: 21A, 28B (n = 49), later- onset phenotype men 2A, 9B, 1C (n = 12) and later- onset phenotype woman 15A, 4B (n = 19).

SARS-CoV-2 infection was defined as positive naso-pharyngeal PCR testing or/and presence of serum SARS-CoV-2 IgG antibodies.

The course of the COVID-19 severity was characterized as asymptomatic, mild, moderate, severe or critical (World Health Organization- WHO categories), according to National Institute of Health (NIH) recommendations [12].

Additionally, the following symptoms were recorded: fever, cough- dry, shortness of breath, cough (productive or dry), sore throat, anosmia, ageusia, myalgias/body aches, lethargy, diarrhea, rash, conjunctivitis, COVID-19 toes/ fingers, abdominal pain, pain crisis or other (including generalized weakness, chest pain, headache, dry throat, non-specific). As “long COVID “ symptoms, the following symptoms were evaluated (if novel or increased post-infection): extreme tiredness (fatigue), shortness of breath, chest pain or tightness, problems with memory and concentration ("brain fog"), insomnia, dizziness, pins and needles, joint pain, depression and anxiety, tinnitus, earaches, feeling sick, diarrhea, stomach aches, loss of appetite, a fever, cough, headaches, changes to sense of smell or taste and rashes, lasting for 4 weeks or more after the start of acute COVID-19, as suggested by the National Institute for Health and Care Excellence (NICE) guidelines [13].

The risk factors for severe COVID-19 were extracted from medical records and divided in FD-related and non-FD-related organ manifestations, as summarized in Table 1. As non-FD-related organ manifestations, all risk factors for severe COVID-19, as suggested by CDC, were included. More specifically, we screened medical records regarding cancer, chronic liver or lung disease, dementia or other neurological conditions, diabetes mellitus, obesity, blood disorders, smoking (current or former), non-FD related vascular disease, tuberculosis, all before the SARS-Co2 infection [14]. Age was considered a FD-related risk factor, because of shorter life-expectancy of patients with FD, compared to the general population [15]_._

## Results

Among the 104 FD patients (24 classic and 12 later- onset phenotype men and 49 classic and 19 later-onset phenotype women) regularly followed by our specialized center during the pandemic, 13/104 (12.5%) were diagnosed with SARS-CoV-2 infection and, more specifically, 16.7% of men (4/24) and 12.2% of women of classic phenotype and 25% of the men of later-onset phenotype (3/12). No female with later-onset FD was diagnosed with SARS-CoV-2 infection (0%, 0/8). Regarding risk stratification according to Table 1, the infected patients belonged to the following categories: A 38.5% (5/13: 1 man and 4 women of classic phenotype), B 46.2% (6/13, 2 classic and 2 later-onset men, 2 women of classic phenotype) and C 15.4% (2/13, both men, one of classic and one of later-onset phenotype, both in early 50 s).

The detailed information regarding genotype, phenotype, risk factors (FD-related and -unrelated), COVID-19 manifestations, management and outcome of the patients are reported in Table 2 for males and Table 3 females.

**Table 2:** COVID-19 cases reported in male patients with FD

Patient	Age (years)	FD genotype and phenotype	ERT (type, years)	FD risk factors (RC)	Non-FD related risk factors	COVID-19 manifestations	COVID-19 severity according to WHO	COVID-19 management	Acute COVID-19 duration (days)	Long-COVID-19 (duration and symptoms)	Outcome
1 a	22	p.F248L/ Classic	Agalsidase-β, 9y	None (RC A)	Smoking (current)	Anosmia, ageusia, dry cough	Mild	None	5–7	None	Recovered
2 a	45	p.S345P/ Classic	Agalsidase-α, 20y	CKD-StI, LVH, arrhythmias (RC B)	Hypertension	cough, fever (mild), fatigue, headache	Mild	Ibuprofen and Paracetamol	8–10	None	Recovered
3 a	22	p.T412SfsX38/Classic	Agalsidase-β, 15y	mild aortal deficiency (RC B)	Smoking (current) underweight	running nose, headache, fatigue, anosmia	Mild	None	5–7	4-months: anosmia	Recovered
4 a, b	50	p.T412SfsX38/Classic	Agalsidase-α, 16y	Kidney ttransplantation, LVH, mild mitral valve insufficiency, arrhythmias (RC C)	COPD Gold Stadium II, Depression	Phase A: Diarrhoea, fever, pneumonia Phase B: Worsening of pneumonia, Fabry pain crisis, oral candidiasis	Severe	Phase A: Supportive treatment Phase B: Piperacillin/Tazobactam, Dexamethasone, nasal oxygen high-flow, Morphine, Fluconazole	Phase A: 2 days hospitalization-10 days home followed by Phase B: 9 days hospitalization, no ICU	3 months: weight loss, fatigue, lethargy, depressive mood and fear disorder	Recovered
5 a	46	p.F113L/Late-Onset	Agalsidase-β, 13y	LVH (RC B)	Overweight Hyperlipidaemia Hypertension	Arthralgias	Mild	None	3–4	None	Recovered
6 a	51	p.P205S/Late-Onset	Agalsidase-α, 10y	Kidney transplantation LVH arrhythmias (RC C)	Diabetes mellitus Type 2 Asthma Hypertension	Fever, rhinorrhoea, pneumonia	Severe	Nasal oxygen high-flow, more details unknown (hospitalisation in Addis Ababa)	9 days hospitalization, no ICU	None	Recovered
7 b	50	p.R301Q/ Late-Onset	Agalsidase-α, 16y	CKD-StI LVH stroke (RC B)	Overweight Hyperlipidaemia Hypertension	None	Asymptomatic	None	0	None	Recovered

The method of the diagnosis is denoted as a: PCR and b: Abs, denoted in the first column. FD: Fabry Disease; ERT: Enzyme Replacement Therapy; y: years; RC: Risk Category according to Table 1; LVH: Left ventricular hypertrophy; COPD: Chronic obstructive pulmonary disease; CKD-St: Chronic Kidney Disease Stadium. Age is presented as range for anonymization purposes

**Table 3:** COVID-19 cases reported in female patients with FD

Patient	Age (years)	FD genotype and phenotype	ERT (type, years)	FD risk factors (Risk category)	Non-FD related risk factors	COVID-19 manifestations	COVID-19 severity according to WHO	COVID-19 management	Acute COVID-19 duration (days)	Long-COVID-19 (duration and symptoms)	Outcome
8 a	32	p.M42T/Classic	Agalsidase-α, 16y	None (RC A)	Asthma under treatment	Hheadache, loss of appetite, myalgias, arthralgias, fatigue	Mild	Ibuprofen and Paracetamol	3–5	None	Recovered
9 a	68	p.C172R/ Classic	Agalsidase-α, 18y	CKD-StI LVH arrhythmias: pacemaker and anticoagulation (RC B)	Smoking (past) COPD Gold Stadium II Mmoderate sleep apnoea	3-day fever up to 38.3 C, 1.5 days productive cough, myalgias, fatigue	Mild	Paracetamol	4–6	None	Recovered
10 a	39	Functional null allele due to splice site mutation/Classic (Hypermobile Ehlers-Danlos-like syndrome)	Agalsidase-α, 10y	None (RC A)	Ssocial alcohol consumption	Hheadache, dry cough, abdominal acke, dizziness, Fabry pain crisis	Mild	Eemergency department: single morphine administration	14	6-months: fatigue, dry cough and dyspnoea in movement, more frequent pain crisis	Recovered
11 b	31	p.I317T/Classic	Agalsidase-β, 2y	None (RC A)	None	None	Asymptomatic	None	0	None	Recovered
12 b	21	p.S345P/ Classic	Agalsidase-β, 10y	CKD-StI (RC A)	Smoking (past)	None	Asymptomatic	None	0	None	Recovered
13 a,b	46	p.T412SfsX38/Classic	Agalsidase-α, 16y	CKD-StI, Arrhythmias (RC B)	Overweight Smoking (current) Hypercholesterinemia Depression	Myalgias	Mild	Paracetamol	10–12	None	Recovered

The method of the diagnosis is denoted as a: PCR and b: Abs, denoted in the first column. FD: Fabry Disease; ERT: Enzyme Replacement Therapy; y: years; RC: Risk Category according to Table 1; LVH: Left ventricular hypertrophy; COPD: Chronic obstructive pulmonary disease; CKD-St: Chronic Kidney Disease Stadium. Age is presented as range for anonymization purposes

Overall, from the infected patients, 2/13 (15.4%) developed severe COVID-19 with hospitalization and oxygen mask; both were kidney transplant recipients on immunosuppression. The others either developed mild COVID-19 manifestations (8/13, 61.5%) or were asymptomatic (3/13, 23.1%). No patient died from COVID-19 needed intubation or was admitted to an intensive care unit. All non-hospitalized patients received symptomatic treatment such as analgetics and antipyretic, if any. No Covid-specific treatment was administered in hospitalized or non-hospitalized patients as indicated in the management section of Table 2 and 3.

Of 61 asymptomatic- at the time of blool sampling- patients who were screened for SARS-CoV-2 IgG antibodies, five (8.2%) showed significant titers but only 2/5 (40%) of them had COVID-19 symptoms with PCR confirmation in their medical records over the past months.

## Discussion

The impact of SARS-CoV-2 infection in patients with rare diseases such as FD is unknown. To our knowledge, this is the first clinical study, which systematically evaluated SARS-CoV-2 infection in FD patients during pandemic. Overall, 13 of 104 (12.5%) of the patients of our cohort were diagnosed with SARS-CoV-2 infection and, only 2 of 13 (15.4%) of them, both kidney transplant recipients, developed severe COVID-19. However, no patient died or was admitted an Intensive Care Unit (ICU).

The results of this study contribute to the discussion of risk-adjusted management of FD patients during the pandemic. The debate about the true incidence of SARS-CoV-2 infection among patients with FD is still ongoing, and it is unknown, whether FD constitutes a risk factor for severe COVID-19. Mechanistically, SARS-CoV-2 may require *GLA *which is abundantly expressed in upper and lower airway epithelial cells [16, 17], as an interaction partner in its life cycle and for the membrane fusion when entering the host cell [18]. Along the same lines, previous questionnaire-based studies found low incidence of SARS-CoV-2 infection among patients with FD (0/129 patients and 0/102 respectively) [19, 20]. However, both these studies were based on self- reports of the patients and were focused on the first pandemic wave, so that, in comparison to the present study which covers the whole pre-vaccination pandemic time, no laboratory confirmations took place from the study groups [19, 20]. A relatively low COVID-19 incidence in FD may also be due to avoidance of exposure by shifting in home office, physical distancing, masking and hand- hygiene. Also independently of the pandemic, FD patients tend to reduce social contacts because of acroparesthesia, fatigue and/or dyspnea in response to cardiac and lung involvement.

Other theories regarding mortality and morbidity risk in FD secondary to COVID-19 infection propose that the pathophysiological background of FD may partly play a “protective role” against the cascade of systemic inflammation and organ damage, triggered by the virus [21]. Suggested mechanisms include that the ACE-II receptor, which is the host cell receptor for SARS-CoV-2, is genetically regulated by chromosome X, and may be regulated in a different way in patients with FD [22], or that the dysfunction of lysosomes in FD may interfere with the cellular entry of SARS-CoV-2 [23, 24]. Overall, since the role of ACE-II receptor in SARS-CoV-2 infection is still controversial, the above-mentioned theories remain to be further studied.

Regarding COVID-19 severity in patients with FD, on the one hand and despite the pulmonary pathology of FD [25], clinical case reports from high incidence countries suggest that these patients may experience less severe COVID-19 manifestations [11]. One the other hand, common disease mechanisms of COVID-19 and FD in the end- target organs could result in susceptibility to severe COVID-19, as it has been recently highlighted by international expert recommendations [11]. Hence, especially for the maximum risk for severe COVID-19 (risk category C) we could confirm in our study the expert recommendations, that the transplant recipients are the patients of the highest risk. Overall, the genetic and pathophysiological background of FD may imply a different response of the patients against SARS-CoV2 infection, which needs to be further investigated. Other researchers assumed that SARS-CoV-2 infection may increase the risk for stroke [26] due to the FD pathophysiology involving vascular endothelial dysfunction and activation of chronic inflammatory cascades leading to a pro-thrombotic state in addition to the pathophysiology of COVID-19 [21]. Such a hypothesis has not been confirmed in our study nor by the previous study of Laney and coworkers [11].

Importantly, two male patients in their early 50 s, one classic and one later onset phenotype, both kidney transplant recipients on immunosuppression and both suffering from cardiac disease, experienced severe COVID-19 and needed hospitalization. The other patients were either mildly diseased or asymptomatic, so that nobody died or required ICU admission or COVID-19 specific treatment. Our observations are in line with the few previously reported cases of patients with FD infected with SARS-CoV-2 [11]. Laney et al. reported that two of 22 SARS-CoV-2 infected FD patients died. One of them, a classic male in late 50 s, kidney transplant recipient, with left ventricular hypertrophy (LVH), stroke and arrhythmias with implanted pacemaker, the other, classic female in mid-60 s with, LVH and arrhythmias. The other 20/22 patients experienced mild or moderate COVID-19 and those hospitalized were either transplant recipients or older than 65 [11]. In another study, two FD patients with kidney and lung involvement also suffered from mild COVID-19 symptoms [27]. Similarly, another death was reported in a 67-year-old male kidney transplant recipient on immunosuppression and with diabetes mellitus [28]. Overall, immunosuppression in the context of organ transplantation, rather than FD itself, seems to represent the most critical risk factor for a severe course of COVID-19 infection.

Our finding, that kidney transplant recipients suffered most from severe COVID-19 is of particular importance regarding vaccination strategies. More specifically, it is known that the different immunosuppressive medications impair or suppress the response to vaccination via multiple mechanisms [29]. Especially for vaccination against SARS-CoV-2 in studies conducted recently in kidney transplant recipients, it has been shown that the vaccine immunogenicity can be reduced [30, 31]. Interestingly, a study conducted in 120 organ transplant recipients reveals that the protective antibody titre can be partially increased when a third and or more booster dosages are administered [32]. Of note, as indicated from Prendecki et al., mRNA vaccines elicit stronger immune responses compared to adenovirus- based vector vaccines in kidney transplant recipients [33]. All the above-mentioned recent findings should be taken into consideration from the treating physician for the preventive measures and the vaccination strategy of patients with FD and organ transplantation.

The limitations of our study include single center data and a relatively small size of the patient cohort due to the nature of the rare disease. Considering also the small number of patients who were infected during the study period (n = 13) the conclusions of the current study need further confirmation. Additionally, SARS-CoV-2 antibodies could only be measured in a subset of FD patients (61 of 104) because the remaining patients did not attend the hospital during this pre-vaccination pandemic period and were consulted by phone. Thus, some asymptomatic COVID-19 infections in these FD patients could have been missed. Additionally, no anti-NP supplementary testing was availablein the IgG seropositive patients. Furthermore, no information on virus variance was available for the infected patients. Furthermore, SARS-CoV-2 antibody titers are known to decrease over time after the infection, which potentially further decreases the sensitivity for the detection of a past asymptomatic COVID-19 infection in our patients.

The strengths of this study is a complete follow-up and a representative observational pre-vaccination period, where gold standard for COVID-19 infection diagnosis was applied and hard clinical outcomes were reported in a genetically and clinically well-defined cohort of FD patients. The complete follow-up is possible due to Swiss regulation that the disease-specific therapies prescription and patients’ follow-up remains reserved for the Fabry Centre. Thus, helpful information on our experience may improve clinical management and patients counselling.

## Conclusions

In summary, the risk for severe COVID-19 in FD patients seems to be driven, like in general population, by the immune system rather than by FD itself. The immunosuppression in kidney transplant recipients represented the highest risk in this population. Further studies on the lysosomal dysfunction in FD and SARS-CoV-2 infection could reveal potential protective or modified responses in those patients and could be of interest for future treatments. However, future clinical studies should focus on the protective effects and safety of the COVID-19 vaccination in patients with FD. As an outlook, the present study could add to recommendations on prevention and management strategies for patients with FD during the ongoing pandemic. FD patients at risk should be protected by vaccination, wearing mask, by social distancing and home office, hand disinfection. Patients should be monitored in case of symptomatology and/or confirmation of SARS-CoV-2 infection.

## Supplementary Information

**Additional file 1:** **SupplementaryTable (S1). **Genotypes of the included patients.

## Data Availability

Additional information or supporting material would be available by the authors upon reasonable request.

## Abbreviations

FD: Fabry Disease
GLA: α-Galactosidase A
COVID-19: Coronavirus disease 2019
Gb3: Globotriaosylceramide
Lyso-Gb3: Globotriaosylsphingosine
ERT: Enzyme-replacement therapy
LVH: Left ventricular hypertrophy
COPD: Chronic obstructive pulmonary disease
CKD: Chronic kidney disease stadium
TIA: Transient Ischemic Attack
WHL: White Matter Lession

## References

[1] Perico L, Benigni A, Casiraghi F, Ng LFP, Renia L, Remuzzi G (2021). Immunity, endothelial injury and complement-induced coagulopathy in COVID-19. Nat Rev Nephrol.

[2] Nowak A, Mechtler TP, Hornemann T, Gawinecka J, Theswet E, Hilz MJ (2018). Genotype, phenotype and disease severity reflected by serum LysoGb3 levels in patients with Fabry disease. Mol Genet Metab.

[3] Mehta A, Ricci R, Widmer U, Dehout F, Garcia de Lorenzo A, Kampmann C (2004). Fabry disease defined: baseline clinical manifestations of 366 patients in the Fabry Outcome Survey. Eur J Clin Invest.

[4] Nowak A, Huynh-Do U, Krayenbuehl PA, Beuschlein F, Schiffmann R, Barbey F (2020). Fabry disease genotype, phenotype, and migalastat amenability: Insights from a national cohort. J Inherit Metab Dis.

[5] Moreno-Martinez D, Aguiar P, Auray-Blais C, Beck M, Bichet DG, Burlina A (2021). Standardising clinical outcomes measures for adult clinical trials in Fabry disease: a global Delphi consensus. Mol Genet Metab.

[6] Arends M, Wanner C, Hughes D, Mehta A, Oder D, Watkinson OT (2017). Characterization of Classical and Nonclassical Fabry Disease: A Multicenter Study. J Am Soc Nephrol.

[7] Oliveira JP, Ferreira S (2019). Multiple phenotypic domains of Fabry disease and their relevance for establishing genotype-phenotype correlations. Appl Clin Genet.

[8] Wanner C, Arad M, Baron R, Burlina A, Elliott PM, Feldt-Rasmussen U (2018). European expert consensus statement on therapeutic goals in Fabry disease. Mol Genet Metab.

[9] Wanner C, Feldt-Rasmussen U, Jovanovic A, Linhart A, Yang M, Ponce E (2020). Cardiomyopathy and kidney function in agalsidase beta-treated female Fabry patients: a pre-treatment vs. post-treatment analysis. ESC Heart Fail..

[10] Germain DP, Elliott PM, Falissard B, Fomin VV, Hilz MJ, Jovanovic A, et al. The effect of enzyme replacement therapy on clinical outcomes in male patients with Fabry disease: A systematic literature review by a European panel of experts. Mol Genet Metab Rep. 2019;19. 10.1016/j.ymgmr.2019.100454 PMC6365982 30775256

[11] Laney DA, Germain DP, Oliveira JP, Burlina AP, Cabrera GH, Hong GR (2020). Fabry disease and COVID-19: international expert recommendations for management based on real-world experience. Clin Kidney J.

[12] Clinical Spectrum of SARS-CoV-2 Infection [Internet]. 2020.

[13] Venkatesan P (2021). NICE guideline on long COVID. Lancet Respir Med.

[14] People with Certain Medical Conditions [Internet]. Available at: https://www.cdc.gov/coronavirus/2019-ncov/need-extra-precautions/people-with-medical-conditions.html. 2020.

[15] Waldek S, Patel MR, Banikazemi M, Lemay R, Lee P (2009). Life expectancy and cause of death in males and females with Fabry disease: findings from the Fabry Registry. Genet Med.

[16] Brown LK, Miller A, Bhuptani A, Sloane MF, Zimmerman MI, Schilero G (1997). Pulmonary involvement in Fabry disease. Am J Respir Crit Care Med.

[17] Franzen D, Haile SR, Kasper DC, Mechtler TP, Flammer AJ, Krayenbuhl PA (2018). Pulmonary involvement in Fabry disease: effect of plasma globotriaosylsphingosine and time to initiation of enzyme replacement therapy. BMJ Open Respir Res.

[18] Kadam SB, Sukhramani GS, Bishnoi P, Pable AA, Barvkar VT (2021). SARS-CoV-2, the pandemic coronavirus: molecular and structural insights. J Basic Microbiol.

[19] Sechi A, Macor D, Valent S, Da Riol RM, Zanatta M, Spinelli A (2020). Impact of COVID-19 related healthcare crisis on treatments for patients with lysosomal storage disorders, the first Italian experience. Mol Genet Metab.

[20] Riccio E, Pieroni M, Limoneglli G, Pisani A (2020). Impact of COVID-19 pandemic on patients with Fabry disease: an Italian experience. Mol Genet Metab.

[21] Parasher A (2021). COVID-19: current understanding of its pathophysiology, clinical presentation and treatment. Postgrad Med J.

[22] Gomez-Lujan M, Cruzalegui C, Aguilar C, Alvarez-Vargas M, Segura-Saldana P. When Frequent (Pandemic) Occurs in a Non-Frequent Disease: Covid-19 and Fabry Disease: Report of Two Cases. Jpn J Infect Dis. 2020. 10.7883/yoken.JJID.2020.729 33250492

[23] Ghosh S, Dellibovi-Ragheb TA, Kerviel A, Pak E, Qiu Q, Fisher M, et al. beta-Coronaviruses use lysosomes for egress instead of the biosynthetic secretory pathway. Cell. 2020;183(6):1520-+. 10.1016/j.cell.2020.10.039 PMC7590812 33157038

[24] Guo C, Tsai SJ, Ai Y, Li M, Pekosz A, Cox A, et al. The D614G Mutation Enhances the Lysosomal Trafficking of SARS-CoV-2 Spike. bioRxiv. 2020.

[25] Franzen DP, Nowak A, Haile SR, Mottet D, Bonani M, Dormond O (2017). Long-term follow-up of pulmonary function in Fabry disease: A bi-center observational study. PLoS ONE.

[26] Reisin RC, Rozenfeld P, Bonardo P (2020). Fabry disease patients have an increased risk of stroke in the COVID-19 ERA. A hypothesis. Med Hypotheses.

[27] Gomez-Lujan M, Cruzalegui C, Aguilar C, Alvarez-Vargas M, Segura-Saldana P (2021). When Frequent (Pandemic) Occurs in a Non-Frequent Disease: COVID-19 and Fabry Disease: Report of Two Cases. Jpn J Infect Dis.

[28] Mahoney R, Lee GK, Zepeda JP, Gabriel C, Hall K, Edwards R (2021). Severe manifestations and treatment of COVID-19 in a transplanted patient with Fabry disease. Mol Genet Metab Rep.

[29] Akkaya M, Kwak K, Pierce SK (2020). B cell memory: building two walls of protection against pathogens. Nat Rev Immunol.

[30] Danthu C, Hantz S, Dahlem A, Duval M, Ba B, Guibbert M (2021). Humoral response after SARS-CoV-2 mRNA vaccination in a cohort of hemodialysis patients and kidney transplant recipients. J Am Soc Nephrol.

[31] Miele M, Busa R, Russelli G, Sorrentino MC, Di Bella M, Timoneri F (2021). Impaired anti-SARS-CoV-2 humoral and cellular immune response induced by Pfizer-BioNTech BNT162b2 mRNA vaccine in solid organ transplanted patients. Am J Transplant.

[32] Hall VG, Ferreira VH, Ku T, Ierullo M, Majchrzak-Kita B, Chaparro C (2021). Randomized trial of a third dose of mRNA-1273 vaccine in transplant recipients. N Engl J Med.

[33] Prendecki M, Thomson T, Clarke CL, Martin P, Gleeson S, De Aguiar RC (2021). Immunological responses to SARS-CoV-2 vaccines in kidney transplant recipients. Lancet.

