# Supplementary material for: COVID-19 in Fabry disease: a reference center prospective study
Source: Orphanet J Rare Dis. 2022 Jun 28;17:250. doi: 10.1186/s13023-022-02386-7 (PMC9237963; doi:10.1186/s13023-022-02386-7)
Supplement: Supplementary file 1 — Additional file 1: SupplementaryTable (S1). Genotypes of the included patients. [file 13023_2022_2386_MOESM1_ESM.docx]

| Age | Mutation | Predicted enzyme protein change |
| --- | --- | --- |
| Classic Phenotype, men (n=24) | | |
| 57 | Deletion exon 2  (g2962, g5871) |  |
| 39 | c.125T>C | p.M42T |
| 42 | c.125T>C | p.M42T |
| 30 | c.125T>C | p.M42T |
| 46 | c.136C>T | p.H46Y |
| 49 | c.370-2A>G  (IVS2-2A>G) | Functional null allele due to splice site mutation |
| 51 | c.370-2A>G  (IVS2-2A>G) | Functional null allele due to splice site mutation |
| 41 | IVS3+405T>G | Functional null  allele due to  splice site  mutation |
| 30 | c.559_560delAT | p.Met187Valfs*6 |
| 27 | c.581C>A | p.T194N |
| 61 | c.581C>T | p.T194I |
| 74 | c.581C>T | p.T194I |
| 43 | c.581C>T | p.T194I |
| 34 | c.679C>T | p.R227X |
| 22 | c.744_745delTA | p.F248L |
| 20 | c.796G>T | p.D266Y |
| 45 | c.827G>A | p.S276N |
| 59 | c.899T>A | p.L300H |
| 45 | c.1033T>C | p.S345P |
| 48 | c.1033T>C | p.S345P |
| 32 | c.1055_1057dupCTA | p.A352_M353insT |
| 34 | c.1146delCTT | delF383 |
| 22 | c.1235_1236delCT | p.T412SfsX38 |
| 50 | c.1235_1236delCT | p.T412SfsX38 |
| Classic Phenotype, women (n=49) | | |
| 20 | Deletion exon 2  (g2962, g5871) |  |
| 21 | Deletion exon 2  (g2962, g5871) |  |
| 53 | c.72G>A | p.W24X |
| 32 | c.125T>C | p.M42T |
| 32 | c.125T>C | p.M42T |
| 36 | c.125T>C | p.M42T |
| 52 | c.365delA | p.N122IfsX |
| 71 | c.370-2A>G | Functional null  allele due to  splice site  mutation |
| 68 | c.514T>C | p.C172R |
| 38 | c.581C>T | p.T194I |
| 70 | c.581C>T | p.T194I |
| 45 | c.581C>T | p.T194I |
| 71 | c.581C>T | p.T194I |
| 40 | c.581C>T | p.T194I |
| 75 | c.581C>T | p.T194I |
| 26 | c.581C>T | p.T194I |
| 44 | c.581C>T | p.T194I |
| 46 | c.581C>T | p.T194I |
| 30 | c.581C>T | p.T194I |
| 69 | c.581C>T | p.T194I |
| 39 | c.581C>T | p.T194I |
| 33 | c.581C>T | p.T194I |
| 39 | c.640-3C>G | Functional null  allele due to  splice site  mutation |
| 55 | c.680G>A | p.R227Q |
| 33 | c.704C>A | p.S235Y |
| 27 | c.744_745delTA | p.F248L |
| 62 | c.796G>T | p.D266Y |
| 60 | c.796G>T | p.D266Y |
| 30 | c.899T>A | p.L300H |
| 29 | c.901C>T | p.R301X |
| 42 | c.901C>T | p.R301X |
| 28 | c.950T>C | p.I317T |
| 47 | c.1033T>C | p.S345P |
| 21 | c.1033T>C | p.S345P |
| 28 | c.1033T>C | p.S345P |
| 32 | c.1033T>C | p.S345P |
| 58 | c.1033T>C | p.S345P |
| 35 | c.1055_1057dupCTA | p.A352_M353insT |
| 32 | c.1146delCTT | delF383 |
| 37 | c.1167dupT | p.V390CfsX9 |
| 42 | c.1167dupT | p.V390CfsX9 |
| 58 | c.1167dupT | p.V390CfsX9 |
| 24 | c.1167dupT | p.V390CfsX9 |
| 30 | c.1167dupT | p.V390CfsX9 |
| 18 | c.1167dupT | p.V390CfsX9 |
| 74 | c.1167dupT | p.V390CfsX9 |
| 46 | c.1235_1236delCT | p.T412SfsX38 |
| 17 | c.1235_1236delCT | p.T412SfsX38 |
| 69 | c.1235_1236delCT | p.T412SfsX38 |
| Late Onset Phenotype, men (n=12) | | |
| 63 | c.337T>C | p.F113L |
| 46 | c.337T>C | p.F113L |
| 23 | c.337T>C | p.F113L |
| 46 | c.337T>C | p.F113L |
| 51 | c.613C>T | p.P205S |
| 70 | c.644A>G | p.N215S |
| 72 | c.644A>G | p.N215S |
| 66 | c.713G>A | p.S238N |
| 50 | c.902G>A | p.R301Q |
| 43 | c.902G>A | p.R301Q |
| 74 | c.902G>A | p.R301Q |
| 47 | c.1196G>C | p.W399S |
| Late Onset Phenotype, women (n=19) | | |
| 40 | c.337T>C | p.F113L |
| 39 | c.337T>C | p.F113L |
| 35 | c.337T>C | p.F113L |
| 18 | c.337T>C | p.F113L |
| 18 | c.337T>C | p.F113L |
| 31 | c.337T>C | p.F113L |
| 37 | c.337T>C | p.F113L |
| 22 | c.337T>C | p.F113L |
| 60 | c.644A>G | p.N215S |
| 35 | c.644A>G | p.N215S |
| 29 | c.644A>G | p.N215S |
| 68 | c.644A>G | p.N215S |
| 41 | c.713G>A | p.S238N |
| 55 | c.870G>C | p.M290I |
| 44 | c.902G>A | p.R301Q |
| 79 | c.902G>A | p.R301Q |
| 47 | c.902G>A | p.R301Q |
| 23 | c.902G>A | p.R301Q |
| 24 | c.902G>A | p.R301Q |
